# Obstetric outcomes during delivery hospitalizations among obese pregnant women in the United States

**DOI:** 10.1038/s41598-022-10786-9

**Published:** 2022-04-27

**Authors:** Muni Rubens, Venkataraghavan Ramamoorthy, Anshul Saxena, Peter McGranaghan, Emir Veledar, Agueda Hernandez

**Affiliations:** 1 Miami Cancer Institute, Miami, FL USA; 2 Baptist Health South Florida, Miami, FL USA; 3 Department of Internal Medicine and Cardiology, Charité—Universitätsmedizin Berlin, Corporate Member of Freie Universität Berlin and Humboldt Universität Zu Berlin, 10117 Berlin, Germany

**Keywords:** Health care, Medical research, Obesity

## Abstract

The rates of both maternal and fetal adverse outcomes increase significantly with higher body mass index. The aim of this study was to calculate national estimates of adverse maternal and fetal outcomes and associated hospitalization cost among obese pregnant women using a national database. This study was a retrospective analysis of data retrieved from Nationwide Inpatient Sample database, collected during 2010–2014. The primary outcomes of this study were adverse maternal and fetal outcomes, hospital length of stay, and hospitalization cost. There was a total of 18,687,217 delivery-related hospitalizations, of which 1,048,323 were among obese women. Obese women were more likely to have cesarean deliveries (aOR 1.70, 95% CI 1.62–1.79) and labor inductions (aOR 1.51, 95% CI 1.42–1.60), greater length of stay after cesarean deliveries (aOR 1.14, 95% CI 1.08–1.36) and vaginal deliveries (aOR 1.48, 95% CI 1.23–1.77). They were also more likely to have pregnancy-related hypertension, preeclampsia, gestational diabetes, premature rupture of membranes, chorioamnionitis, venous thromboembolism, excessive fetal growth, and fetal distress. Obese pregnant women had significantly greater risk for adverse obstetrical outcomes, which substantially increased the hospital and economic burden. Risk stratification of pregnant patients based on obesity could also help obstetricians to make better clinical decisions and improve patient outcomes.

## Introduction

Among women of reproductive age, the prevalence of obesity increased by nearly 30% during the past decade in the US^1^. The number of women having pre-pregnancy obesity increased from 26.1% in 2016 to 29.0% in 2019^1^. The rates of both maternal and fetal adverse outcomes increase significantly with higher body mass index (BMI). Maternal complications among obese pregnant women include gestational hypertension, preeclampsia, gestational diabetes mellitus, and increased need for cesarean deliveries^2^. Likewise, fetal complications include macrosomia, preterm birth, shoulder dystocia, neonatal death, and stillbirth^2^. For example, in a large-scale multicenter study, there was significant association between obesity and adverse maternal and fetal outcomes such as gestational hypertension (adjusted odds ratios [aOR] 2.5), preeclampsia (aOR, 1.6), gestational diabetes (aOR 2.6), and birth weight ≥ 4000 g (aOR 1.7) and ≥ 4500 g (aOR 2.0)^3^. Similarly, in a large cohort study done among 3247 women with delivery hospitalizations, obese patients were significantly more likely to experience gestational hypertension (aOR 8.6), preeclampsia (aOR 2.1), gestational diabetes (aOR 5.6), dystocia (aOR = 2.1), induced labor (aOR 2.6), failed induction of labor (aOR 18.1), cesarean delivery (aOR 1.8), large-for-gestational-age newborns (aOR 3.7)^4^. In a meta-analysis of 59 studies done by Lutsiv et al., morbid obesity was significantly associated with preterm birth (relative risk [RR], 1.3), large-for-gestational age fetus (RR 1.4), and small-for-gestational age fetus (RR 0.9)^5^. Maternal obesity during pregnancy has long-term negative consequences on the health and vitality of the children. Babies born to obese mothers are at a greater risk of developing obesity and diabetes mellitus in later life^2^. They also have greater risks for neuropsychiatric and cognitive disorders^6^.

There are many small and large-scale studies that examined the effects of obesity on maternal and fetal outcomes. However, studies that used nationally representative hospital databases are scarce. The aim of this study was to calculate national estimates of adverse maternal and fetal outcomes associated with obesity among pregnant women in the US using a nationally representative database. In addition, we also calculated the national estimates of hospitalization cost and for these high-risk deliveries.

## Methods

### Study design and data source

The current study was a retrospective cross-sectional analysis of National Inpatient Sample (NIS) data collected between 2010 and 2014. The NIS constitutes the largest all payer in-patient database in the US and was developed as a part of Healthcare Cost and Utilization Project (HCUP) by the Agency for Healthcare Research and Quality (AHRQ). The NIS collects a 20% stratified sample from all US community hospitals included in the American Hospital Association directory. Each year nearly 35 million weighted discharges are recorded from nearly 1000 hospitals within the US. Discharge weights provided by the NIS can be used to calculate national estimates from the data collected from these hospitals. International Classification of Diseases, Ninth Revision (ICD-9) and Clinical Classification Software (CCS) codes are used for recording the diagnoses and procedures during these hospitalizations. NIS does not have information about medication use. In addition, successive admissions for the same patient cannot be linked because each admission is considered as an independent event and associated identifiers are redacted for confidentiality. Our study was considered exempt from institutional review board approval because NIS data is already deidentified.

### Patient selection

Delivery hospitalizations were identified using ICD-9 codes that were recommended by a previous validation study^7^. From this group we identified obese and non-obese women based on the dichotomous variable already present in the NIS. Obesity was identified as a comorbidity and hence considered pregestational obesity.

### Demographics and hospital characteristics

Demographics and hospital characteristics were extracted directly from the NIS. Past medical history such as hypertension, diabetes mellitus, chronic renal disease, alcohol or substance abuse, depression, and psychiatric disorders, and past obstetric history such as cesarean section and multiple births were identified using ICD-9 codes (Supplementary Table 1).

### Adverse maternal and fetal outcomes

Maternal and fetal outcomes were selected based on previous studies that showed association with obesity^3–5^. We used ICD-9 codes for identifying adverse maternal and fetal outcomes (Supplementary Table 2). Overall maternal morbidity was estimated using the Severe Maternal Morbidity Composite Outcome developed by the Centers for Disease Control and Prevention^8^. Other adverse maternal outcomes included cesarean delivery, induction of labor, pregnancy-related hypertension, eclampsia, antepartum hemorrhage, postpartum hemorrhage, gestational diabetes, preterm labor, premature rupture of membranes, chorioamnionitis, and venous thromboembolism. Adverse fetal outcomes included poor fetal growth, excessive fetal growth, fetal distress, central nervous system malformations, chromosomal abnormalities, hereditary disease in family possible affecting the fetus, decreased fetal movements, and stillbirth.

### Statistical analyses

Demographic and clinical characteristics and adverse maternal and fetal outcomes were compared between obese and non-obese pregnant women using Rao-Scott chi-square test for categorical variables, and t test and Mann–Whitney U test for continuous variables. To overcome indication bias, we created a matched propensity score design that adjusted for the differences in demographic and clinical characteristics between obese and non-obese patients. We created a nonparsimonious multivariable logistic regression model that adjusted for nonrandom treatment selections using propensity scores calculated for all patients and conditioned on demographic and clinical characteristics. We used a 1:1 greedy matching algorithm with a caliper width of 0.25 times the standard deviation of the logit of the propensity score for the purpose of propensity-score matching on the likelihood of being obese versus non-obese. For measured covariates, standardized differences in the distribution of < 10% was considered as adequate match between obese and non-obese groups. After propensity score matching, we compared the outcomes between obese and non-obese women using conditional logistic regression which adjusted for matched pairs.

Hospitalization costs were calculated for all patients by multiplying hospital charges with cost-to-charge ratios. We adjusted hospitalization cost for each year, based on 2014 inflation levels. Since NIS was redesigned in 2012, we used modified discharge weights (“trendwt”) for 2010 and 2011. For this study, we followed the guidelines developed by Khera and Krumholz for using NIS data^9^. All tests were two-sided and statistical significance was set at *P* < 0.05. SAS version 9.4 (SAS Institute, Cary, North Carolina) was used for the analyses.

## Results

### Demographics and clinical characteristics

There was a total of 18,687,217 delivery-related hospitalizations reported during 2010–2014, of which 1,048,323 (5.6%) were among obese women. Differences in demographics and clinical characteristics between obese and non-obese pregnant women are shown in Table 1. The mean age of obese pregnant women was 28.5 years while among non-obese was 28.0 years. Among both groups, majority of pregnant women were white, followed by Hispanic and black. Majority of the patients in the obese group had Medicaid (50.6%), while among non-obese group majority had Private insurance (50.1%) coverages. Majority of the patients in the obese group fell in the lowest income quartile (32.5%), while among non-obese group, income distribution was relatively homogenous across the 4 income quartiles. A number of pre-existing conditions considered as high risk for adverse delivery outcomes such as multiple births, previous cesarean deliveries, diabetes mellitus, hypertension, chronic renal disease, liver disease, chronic pulmonary disease, depression, alcohol or substance abuse, and psychiatric disorders were significantly higher among obese pregnant women. All demographic and clinical characteristics differed significantly between obese and non-obese pregnant women undergoing delivery hospitalizations. After propensity score matching, there were no significant differences in any demographic and clinical characteristic, except for diabetes mellitus (*P* < 0.001) (Table 1).

**Table 1:** Baseline clinical characteristics of obese and non-obese pregnant women.

Characteristic	Not matched		*P* value	Matched		*P* value
	Obese (n = 1,048,323)	Non-Obese (n = 17,638,894)		Obese (n = 972,620)	Non-Obese (n = 970,175)	
Age in years, mean (SE)	28.5 (0.03)	28.0 (0.03)	< 0.001	28.2 (0.03)	28.2 (0.03)	0.602
*Race, % (SE)*			< 0.001			0.050
White	47.2% (0.6)	53.2% (0.5)		46.3% (1.6)	47.5% (1.4)	
Black	23.3% (0.5)	13.9% (0.2)		24.0% (1.6)	23.0% (1.2)	
Hispanic	22.4% (0.5)	21.5% (0.4)		23.6% (1.5)	23.4% (1.2)	
Asian or Pacific Islander	2.1% (0.1)	5.5% (0.2)		1.9% (0.3)	2.4% (0.4)	
Native American	1.1% (0.1)	0.78% (0.1)		0.9% (0.1)	0.5% (0.1)	
Other	3.6% (0.2)	4.8% (0.2)		3.3% (0.4)	3.2% (0.3)	
*Insurance type, % (SE)*			< 0.001			0.229
Medicare	1.3% (0.05)	0.65% (0.0)		1.2% (0.2)	1.0% (0.2)	
Medicaid	50.6% (0.5)	43.4% (0.4)		49.7% (1.6)	50.1% (1.2)	
Private insurance	44.0% (0.6)	50.1% (0.4)		45.2% (1.7)	45.3% (1.3)	
Self-pay	1.4% (0.1)	2.6% (0.1)		1.4% (0.2)	1.4% (0.1)	
No charge^a^	0.08% (0.0)	0.12% (0.0)		0.2% (0.1)	0.1% (0.1)	
Other^b^	2.4% (0.1)	2.9% (0.1)		2.4% (0.3)	2.0% (0.2)	
*Median household income for patient’s zip code, % (SE)*			< 0.001			0.489
Quartile 1	32.5% (0.6)	27.4% (0.4)		32.5% (1.7)	33.7% (1.4)	
Quartile 2	26.5% (0.3)	24.9% (0.3)		24.9% (0.8)	24.6% (0.8)	
Quartile 3	24.7% (0.3)	25.1% (0.3)		25.9% (1.0)	25.1% (0.8)	
Quartile 4	16.0% (0.4)	22.4% (0.5)		16.6% (1.3)	16.6% (1.2)	
Elective admission, % (SE)	47.6% (0.9)	50.0% (0.8)	< 0.001	47.3% (2.5)	47.1% (2.1)	0.852
Multiple births, % (SE)	2.3% (0.0)	1.8% (0.01)	< 0.001	2.4% (0.1)	2.1% (0.1)	0.014
Previous cesarean delivery, % (SE)	26.4% (0.1)	16.6% (0.1)	< 0.001	26.4% (0.3)	26.7% (0.3)	0.395
Preexisting diabetes mellitus, % (SE)	4.4% (0.1)	0.82% (0.0)	< 0.001	4.5% (0.2)	3.9% (0.1)	< 0.001
Chronic renal disease, % (SE)	0.56% (0.0)	0.25% (0.0)	< 0.001	0.6% (0.0)	0.5% (0.0)	0.050
Preexisting hypertension, % (SE)	10.4% (0.1)	1.8% (0.01)	< 0.001	10.4% (0.4)	10.4% (0.3)	0.877
Depression, % (SE)	5.1% (0.1)	2.1% (0.0)	< 0.001	4.8% (0.2)	4.6% (0.2)	0.338
Alcohol or substance abuse, % (SE)	2.3% (0.1)	1.7% (0.0)	< 0.001	2.1% (0.2)	1.9% (0.1)	0.133
Psychiatric disorders^c^, % (SE)	5.3% (0.1)	2.3% (0.0)	< 0.001	4.5% (0.2)	4.4% (0.2)	0.492
Liver disease, % (SE)	0.3% (0.0)	0.1% (0.0)	< 0.001	0.3% (0.0)	0.2% (0.0)	0.084
Chronic pulmonary disease, % (SE)	9.4% (0.1)	3.5% (0.1)	< 0.001	9.1% (0.3)	9.4% (0.3)	0.281

^a^Care provided as charity, courtesy, or free of charge.

^b^This category includes Worker’s Compensation, the Civilian Health and Medical Program of the Uniformed Services, the Civilian Health and Medical Program of the Department of Veterans Affairs, Title V, and other government programs.

^c^Includes anxiety, adjustment, eating, mood, personality, and psychotic disorders.

### Hospital characteristics

Table 2 shows differences in hospital characteristics between obese and non-obese pregnant women who were hospitalized for delivery. Majority of the patients in both groups were admitted to large, urban hospitals in the south. However, among obese group, majority were admitted to non-teaching hospitals, while among non-obese group, majority were admitted to teaching hospitals. Table 2 also shows comparison of hospital characteristics between the groups after propensity score matching.

**Table 2:** Hospital characteristics of obese and non-obese pregnant women with delivery hospitalizations.

Characteristics	Not matched		*P* value	Matched		*P* value
	Obese (n = 1,048,323)	Non-Obese (n = 17,638,894)		Obese (n = 972,620)	Non-Obese (n = 970,175)	
*Region, % (SE)*			< 0.001			0.948
Northeast	14.0% (0.72)	16.1% (0.42)		14.2% (2.1)	13.9% (1.0)	
Midwest	19.0% (0.62)	21.2% (0.47)		12.7% (1.3)	12.7% (0.9)	
South	38.0% (0.96)	38.1% (0.71)		41.0% (2.5)	42.1% (1.7)	
West	28.8% (1.02)	24.4% (0.63)		32.2% (2.6)	31.3% (1.5)	
*Location, % (SE)*			< 0.001			0.932
Rural	9.0% (0.69)	11.5% (0.48)		8.3% (0.7)	8.3% (0.4)	
Urban	90.9% (0.69)	88.4% (0.48)		91.7% (0.7)	91.7% (0.4)	
*Bed size*^*a*^*, % (SE)*			0.133			0.945
Small	11.8% (0.49)	12.4% (0.32)		9.4% (1.2)	9.1% (0.7)	
Medium	27.3% (0.84)	28.2% (0.58)		25.5% (2.1)	25.4% (1.4)	
Large	60.8% (0.94)	59.3% (0.64)		65.1% (2.3)	65.5% (1.5)	
*Teaching status, % (SE)*			0.012			0.898
Teaching	47.4% (2.39)	53.0% (1.4)		52.1% (2.6)	52.3% (1.6)	
Non-teaching	52.5% (2.39)	46.9% (1.4)		47.9% (2.6)	47.7% (1.6)	

^a^Bed size categories are based on hospital beds and are specific to the hospital's location and teaching status. For details of categorization, please see: https://www.hcup-us.ahrq.gov/db/vars/hosp_bedsize/nisnote.jsp.

### Maternal and fetal outcomes

Table 3 shows differences in obstetrical outcomes between obese and non-obese pregnant women who were hospitalized for delivery. All adverse obstetrical outcomes were significantly higher among obese pregnant women, except, antepartum hemorrhage (*P* = 0.956) and poor fetal growth (*P* = 0.155). After propensity score matching, majority of the adverse obstetrical outcomes remained significantly higher among obese women, except for maternal death (*P* = 0.443), preterm labor (*P* = 0.935), fetal central nervous system malformations (*P* = 0.214), hereditary disease in family possible affecting fetus (*P* = 0.854), and stillbirths (*P* = 0.814), which became non-significant. Hospitalization cost was significantly higher for obese women ($5283 versus $4331, *P* < 0.001). Among elective delivery hospitalizations, 37.1% were cesarean deliveries, while among non-elective delivery hospitalizations, 29.1% were cesarean deliveries (*P* < 0.001).

**Table 3:** Obstetrical outcomes among obese and non-obese pregnant women.

Outcomes	Not matched		*P* value	Matched		*P* value
	Obese (n = 1,048,323)	Non-Obese (n = 17,638,894)		Obese (n = 972,620)	Non-Obese (n = 970,175)	
Maternal death	0.01% (0.002)	0.01% (0.0004)	< 0.001	0.02% (0.0)	0.02% (0.0)	0.443
Severe maternal morbidity	2.5% (0.04)	1.6% (0.01)	< 0.001	2.5% (0.1)	2.3% (0.1)	0.005
Cesarean delivery	52.8% (0.27)	31.9% (0.12)	< 0.001	54.4% (0.8)	40.9% (0.5)	< 0.001
Induction of labor	24.1% (0.23)	18.8% (0.15)	< 0.001	23.7% (0.6)	17.0% (0.4)	< 0.001
*LOS, mean (SE)*						
Cesarean delivery	3.8 (0.02)	3.5 (0.01)	< 0.001	3.8 (0.0)	3.7 (0.1)	0.001
Vaginal delivery	2.5 (0.0)	2.2 (0.0)	< 0.001	2.5 (0.0)	2.3 (0.0)	< 0.001
*Length of stay > 6 days*						
Cesarean delivery	5.3% (0.1)	2.9% (0.0)	< 0.001	5.3% (0.3)	4.7% (0.2)	0.013
Vaginal delivery	1.6% (0.0)	0.6% (0.0)	< 0.001	1.8% (0.1)	1.1% (0.1)	< 0.001
Gestational hypertension	20.7% (0.17)	7.9% (0.05)	< 0.001	20.6% (0.4)	10.3% (0.2)	< 0.001
Preeclampsia	0.11% (0.01)	0.06% (0.001)	< 0.001	0.1% (0.0)	0.1% (0.0)	< 0.001
Antepartum hemorrhage	1.5% (0.03)	1.5% (0.01)	0.956	1.5% (0.1)	1.8% (0.1)	< 0.001
Postpartum hemorrhage	3.7% (0.06)	3.0% (0.04)	< 0.001	3.5% (0.2)	3.1% (0.1)	0.001
Gestational diabetes	15.8% (0.15)	5.9% (0.04)	< 0.001	15.1% (0.4)	6.3% (0.2)	< 0.001
Preterm labor	7.9% (0.11)	6.5% (0.05)	< 0.001	9.1% (0.3)	9.1% (0.2)	0.935
Premature rupture of membranes	4.8% (0.08)	4.2% (0.05)	< 0.001	4.7% (0.2)	4.0% (0.2)	< 0.001
Chorioamnionitis	2.6% (0.06)	1.88% (0.03)	< 0.001	2.6% (0.2)	1.8% (0.1)	< 0.001
Venous thromboembolism	0.51% (0.01)	0.19% (0.003)	< 0.001	0.5% (0.0)	0.3% (0.0)	< 0.001
Poor fetal growth	2.5% (0.05)	2.5% (0.02)	0.155	2.3% (0.1)	2.9% (0.1)	< 0.001
Excessive fetal growth	6.7% (0.10)	2.3% (0.02)	< 0.001	6.9% (0.3)	2.3% (0.1)	< 0.001
Fetal distress	18.5% (0.22)	14.4% (0.14)	< 0.001	17.8% (0.5)	14.3% (0.4)	< 0.001
Central nervous system malformations	0.08% (0.01)	0.05% (0.002)	< 0.001	0.1% (0.0)	0.1% (0.0)	0.214
Chromosomal abnormalities	0.11% (0.01)	0.08% (0.002)	< 0.001	2.4% (0.1)	1.8% (0.1)	< 0.001
Hereditary disease in family possible affecting fetus	0.02% (0.003)	0.01% (0.001)	0.002	0.0% (0.0)	0.0% (0.0)	0.854
Decreased fetal movements	1.2% (0.03)	0.69% (0.01)	< 0.001	1.0% (0.1)	0.7% (0.1)	< 0.001
Stillbirth	0.78% (0.02)	0.60% (0.01)	< 0.001	0.8% (0.0)	0.8% (0.0)	0.814
Hospitalization cost, median (IQR)	5229 (3640–7622)	4075 (2861–5921)	< 0.001	5283 (3649–7686)	4331 (2984–6402)	< 0.001

### Association between obesity and maternal and fetal outcomes

**Figure 1:**
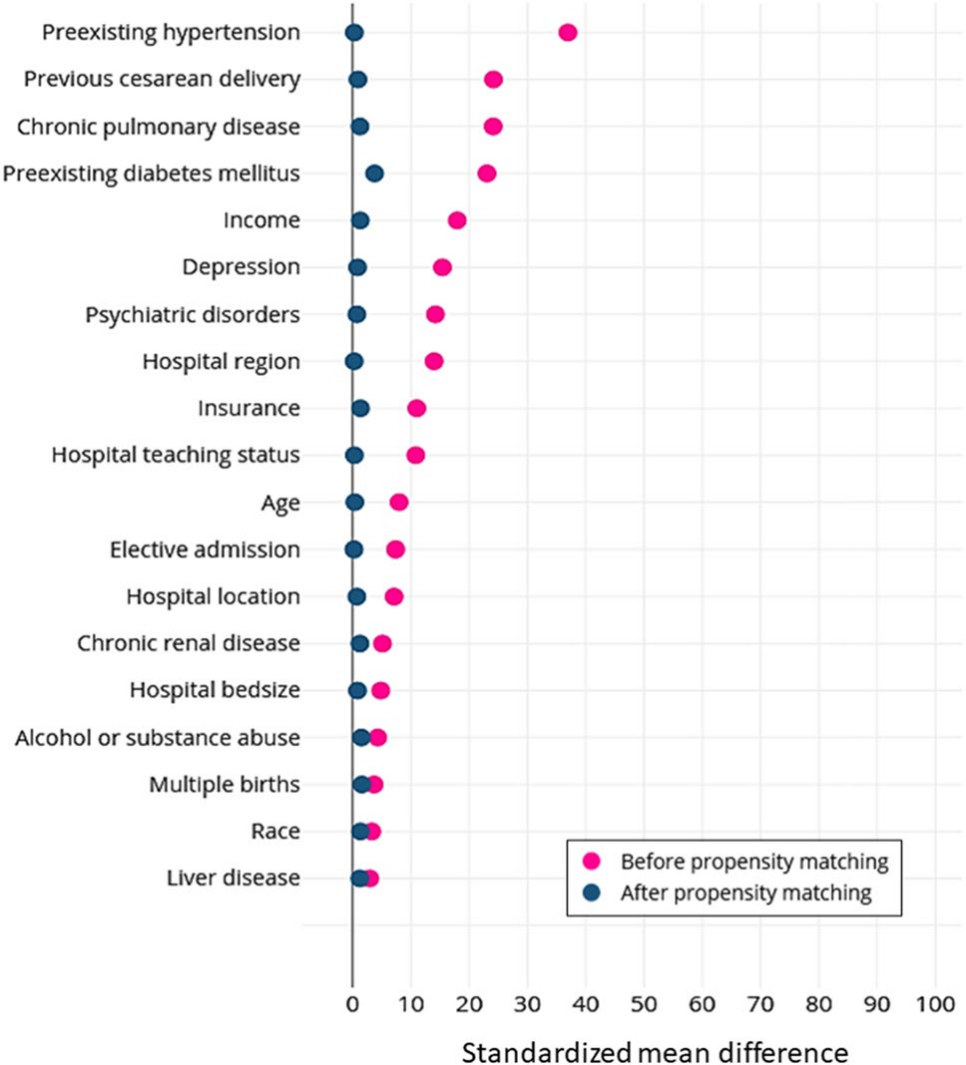
Standardized differences between variables before and after propensity matching for obese versus non-obese women. Note: Vertical lines represent the acceptable range of standardized difference after propensity score matching (0–10%).

**Figure 2:**
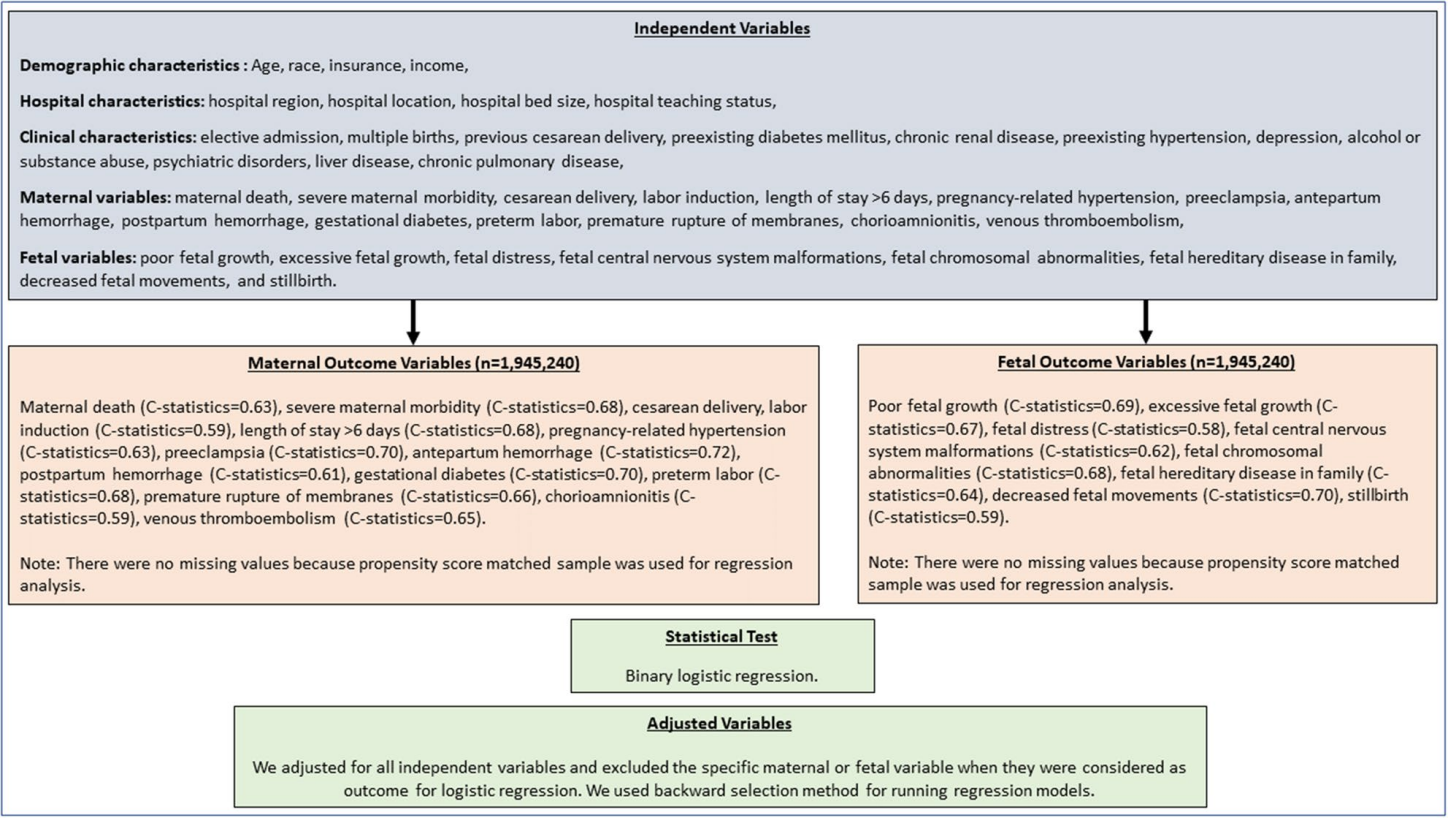
List of independent and dependent variables, c-statistics, and how the variables were controlled for in the regression models.

After propensity matching, the standardized mean difference was less than 10% for all covariates, which indicated that matching was successful in achieving covariate balance between obese and non-obese groups (Fig. 1). In propensity score-matched analysis, obese women were more likely than non-obese women to have cesarean deliveries (adjusted odds ratio [aOR] 1.70, 95% CI 1.62–1.79) and labor inductions (aOR1.51, 95% CI 1.42–1.60) (Table 4). Obese women were more likely to have greater length of stay after cesarean deliveries (aOR 1.14, 95% CI 1.08–1.36) and vaginal deliveries (aOR 1.48, 95% CI 1.23–1.77). Obese women were more likely to have risk factors for adverse obstetrical outcomes such as pregnancy-related hypertension (aOR 2.17, 95% CI 2.06–2.29), preeclampsia (aOR 2.06, 95% CI 1.42–2.99), gestational diabetes (aOR 2.75, 95% CI 2.60–2.90), premature rupture of membranes aOR 1.17, 95% CI 1.08–1.27]), chorioamnionitis (aOR 1.39, 95% CI 1.25–1.55), and venous thromboembolism (aOR 1.63, 95% CI 1.34–1.99). Obese women were more likely to have adverse fetal outcomes such as excessive fetal growth (aOR 3.18, 95% CI 2.96–3.43) and fetal distress (aOR 1.28, 95% CI 1.21–1.35). However, obese women were less likely to experience adverse fetal outcomes such as poor fetal growth (aOR 0.75, 95% CI 0.68–0.82). Figure 2 shows the independent and dependent variables, c-statistics, and how the variables were controlled for in the regression models.

**Table 4:** Association between obesity and adverse obstetrical outcomes among women who were hospitalized for delivery.

Outcomes	aOR (95% CI)^a^
*Maternal outcomes*	
Maternal death	0.95 (0.37–2.47)
Severe maternal morbidity	0.89 (0.81–1.17)
Cesarean delivery	**1.70 (1.62–1.79)**
Labor induction	**1.51 (1.42–1.60)**
*Length of stay > 6 days*	
Cesarean delivery	**1.14 (1.08–1.36)**
Vaginal delivery	**1.48 (1.23–1.77)**
Pregnancy-related hypertension	**2.17 (2.06–2.29)**
Preeclampsia	**2.06 (1.42–2.99)**
Antepartum hemorrhage	0.78 (0.71–1.25)
Postpartum hemorrhage	1.07 (0.98–1.16)
Gestational diabetes	**2.75 (2.60–2.90)**
Preterm labor	0.95 (0.90–1.01)
Premature rupture of membranes	**1.17 (1.08–1.27)**
Chorioamnionitis	**1.39 (1.25–1.55)**
Venous thromboembolism	**1.63 (1.34–1.99)**
*Fetal outcomes*	
Poor fetal growth	**0.75 (0.68–0.82)**
Excessive fetal growth	**3.18 (2.96–3.43)**
Fetal distress	**1.28 (1.21–1.35)**
Fetal central nervous system malformations	1.32 (0.86–2.03)
Fetal chromosomal abnormalities	0.90 (0.61–1.34)
Fetal hereditary disease in family possible affecting fetus	1.00 (0.48–2.08)
Decreased fetal movements	**1.44 (1.26–1.63)**
Stillbirth	0.95 (0.83–1.09)

^a^Age, race, insurance, income, hospital region, hospital location, hospital bed size, hospital teaching status, elective admission, multiple births, previous cesarean delivery, preexisting diabetes mellitus, chronic renal disease, preexisting hypertension, depression, alcohol or substance abuse, psychiatric disorders, liver disease, chronic pulmonary disease, maternal death, severe maternal morbidity, cesarean delivery, labor induction, length of stay > 6 days, pregnancy-related hypertension, preeclampsia, antepartum hemorrhage, postpartum hemorrhage, gestational diabetes, preterm labor, premature rupture of membranes, chorioamnionitis, venous thromboembolism, poor fetal growth, excessive fetal growth, fetal distress, fetal central nervous system malformations, fetal chromosomal abnormalities, fetal hereditary disease in family, decreased fetal movements, and stillbirth.

## Discussion

Obese women were at significantly greater risk than non-obese women for a number of obstetrical complications during delivery hospitalizations. Supporting evidence include higher rates of pregnancy-related hypertension, eclampsia, gestational diabetes, premature rupture of membranes, chorioamnionitis, and venous thromboembolism among obese pregnant women. Obese women were more likely to deliver via cesarean section and undergo labor induction. Obese women also had higher rates of adverse fetal outcomes such as excessive fetal growth and fetal distress. They also had significantly greater hospital stay and higher hospitalization costs. In spite of these adverse findings, we also found that maternal mortality did not differ significantly between the two groups and obese women were at lower risk for poor fetal growth.

The relationship between obesity and adverse obstetrical outcomes has been adequately researched in previous studies. Nevertheless, our findings are important because we used nationally representative data with a large sample size, which helped us to calculate precise national estimates for these outcomes. In our study, we found that obese women were significantly more likely to deliver via cesarean section. However, we were not able to identify the indications for cesarean section in our database. Similar to our findings, in a meta-analysis that looked for the association between obesity and cesarean section, the pooled odds of experiencing caesarean section were more than double among obese women^10^. Similarly, in a large-scale study among 11,922 women, higher BMI was significantly associated with earlier decisions for caesarean section^11^. This study also found that higher BMI was associated with higher rates of oxytocin and epidural analgesia use and decreased use of forceps and vacuum extraction after controlling for confounders. However, in our study we could not explore such specific details due to the limitations in the database used for our study. Studies have also reported that obesity significantly increased surgical, anesthetic, and logistical challenges while planning for caesarean deliveries. For example, obese patients with BMI > 40 kg/m^2^, had longer operation time from beginning of incision to delivery and closure of the surgical wound^12^. Obese pregnant women also had greater incidence of postoperative endometritis, delayed wound healing, wound infection, dehiscence, and greater dosage and duration of antibiotic treatments^13–15^. Our study showed that obese women were significantly at greater risk of receiving labor induction. Similar findings were reported by Wolfe et al., who found that 30% and 34% of women with class I and III obesity received labor induction^16^. A retrospective analysis done among 955 pregnant women showed that higher BMI was associated with longer length of labor induction (*P* = 0.001)^17^. Increased rates of labor induction and caesarean deliveries among obese women could be explained by decreased contractility of the myometrium and prolonged duration of labor^18^. This could be due to higher levels of leptin and cholesterol found in obese individuals. Leptin and cholesterol inhibit calcium influx into the myometrium leading to antagonistic effects against oxytocin, thereby decreasing myometrial contractility^19,20^. These mechanisms lead to clinical manifestations such as prolonged labor, increased need for inductions, and increased incidence of cesarean sections among obese pregnant women.

Our study showed that obese pregnant women were more likely to have obstetric risk factors such as pregnancy-related hypertension and preeclampsia. In a study by Kazemian et al., it was found that obese women had two times greater risk for gestational hypertension^21^. One study also reported that for every 1 kg/m^2^ increase in BMI, there was an associated 6% and 9% increase in the risk for gestational hypertension and preeclampsia, respective^22^. In another study using Stockholm-Gotland Obstetrical database, it was found that the odds of preeclampsia increased proportionally with increasing weight, among overweight and obese pregnant women^23^. These findings could be explained by the fact that obesity constitutes a hyper-inflammatory condition with increased levels of inflammatory markers such as C-reactive protein (CRP) and erythrocyte sedimentation rate (ESR), elevated levels of cytokines such as tumor necrosis factor α (TNF-α), interleukin-6 (IL-6), and IL-8, and higher levels of adhesion molecules such as vascular cell adhesion molecule 1 (VCAM-1), intercellular adhesion molecule 1 (ICAM-1), and P-selectin^24–26^. This heightened inflammatory response increases the release of reactive oxygen species and myeloperoxidase by the inflammatory cells which subsequently attack and destroys the endothelial lining. These mechanisms collectively increase the risk for preeclampsia^27^.

In a meta-analysis by Chu et al., it was found that overweight, obese, and morbidly obese women were at 2-, 4-, and 8-times greater risk for developing gestational diabetes mellitus^28^. In a prospective study that included 256 pregnant women, pre-pregnancy obesity was the strongest and most important predictor of gestational diabetes mellitus^29^. These results were echoed by our study, which also showed that obese women were at significantly greater risk for developing gestational diabetes mellitus. A number of factors such as decreased insulin sensitivity, alterations in glucose, amino acid, and lipid metabolisms, elevated levels of maternal cytokines such as TNF-α, and elevated levels of free fatty acids have been implicated for not only the etiopathogenesis of gestational diabetes mellitus, but also some of its sequalae and complications^30^. Irrespective of these risk factors, it is important to diagnose, monitor, and control gestational diabetes mellitus because it is associated with many maternal and fetal complications such as gestational hypertension and preeclampsia, increased indications for cesarean section, type 2 diabetes in later life, fetal macrosomia, pre-term delivery, fetal hypoglycemia, and stillbirth^31^.

A number of risk factors increasing adverse obstetric outcomes such as premature rupture of membranes, chorioamnionitis, venous thromboembolism, excessive fetal growth, and fetal distress were significantly higher among obese women in our study. Many other studies have reported greater levels of adverse obstetric outcomes as well as associated risk factors among obese pregnant women. A retrospective cohort study that reviewed the records from 11,726 women found that maternal obesity was associated with increased risk of premature rupture of membranes^32^. A secondary analysis of data from a multicenter randomized trial showed that obese women had 60% greater risk for developing chorioamnionitis prior to delivery^33^. In a population-based case control study that included 71,729 women, who had given birth to126,783 children, obesity increased the risk for venous thromboembolism by four times^34^. In a study that included 12,950 deliveries, obese women had significantly greater risk for excessive fetal growth and 2–3 times greater risk for delivering macrosomic babies^35^. Similarly, among 2000 women enrolled in a study, about 20.6% of obese primigravids had fetal distress which necessitated emergency cesarean section^36^. Thus, there is a fairly greater probability that obese women are likely to suffer adverse delivery outcomes during pregnancy. This is reflected in a number of high-risk pregnancies and complicated deliveries as a result of these risk factors. For example, chorioamnionitis increases the risk for, endometritis, blood loss during delivery, and septicemia and thereby greater referrals for cesarean delivery^2^. Premature rupture of membranes increases the risk of bacteremia and pre-term delivery, while venous thromboembolism increases the rates of complications such as pulmonary hypertension, pulmonary embolism, post-thrombotic syndrome, and venous insufficiency^2^. Similarly, excessive fetal growth, which is common among obese women, increases the risk for birth trauma, shoulder dystocia, brachial plexus injury, and meconium aspiration, while fetal distress increases the risk for emergency cesarean delivery^2^. Though the reason for these complications among obese pregnant women are not well understood, factors such as hyperinflammatory states, altered vasoregulatory and clotting mechanisms, disturbances in glucose and lipid metabolisms and other alterations in normal physiological homeostatic processes could be responsible^37–40^.

We also found that hospital length of stay, and hospitalization costs were significantly higher among obese pregnant women. This could be explained by the greater need for advanced treatment and care required for managing the complications. Given the additional health and economic burden posed by these complications, it is crucial that weight control measures should be instituted at the earliest in this population. Though there are no consensus on optimal weight gain during pregnancy, the Institute of Medicine’s recommended weight gain of 15–25 lb for overweight women, and 11–20 lb for obese women should be followed^41^. Obese women should receive preconception counseling about maternal and fetal complications associated with increased body weight. This counseling should also include advise about healthy diet and exercise, and behavioral and lifestyle modifications. Two commonly recommended diets include the Mediterranean diet and the low glycemic load diet. Extremely obese women should consult for bariatric surgeries because research has shown significant reduction in adverse obstetrical outcomes and associated risk factors in pregnancies planned after 12–18 months after these surgeries^42^. Care should also be taken to avoid pregnancies during the weight loss phase after bariatric surgeries and vitamin deficiencies should be promptly corrected before pregnancy^43^. These strategies could help in decreasing the rates and severities of adverse obstetrical outcomes in this population.

The findings in our study have many maternal, fetal, and public health implications. Maternal obesity represents an important public health concern having significant consequences on the health outcomes related to prenatal and postnatal care, and fetal outcomes. We believe that our findings could have significant impact on how obese pregnant women are counseled about the risks of pregnancy and related complications during pregnancy that are associated with obesity. Perinatal counseling among these women could incorporate the caution as well as preemptive considerations for these increased risks. Furthermore, the postpartum period could also be an opportunity where the impact of obesity on other health outcomes besides pregnancy, as well as on physiological changes after delivery and future pregnancies could be communicated to the individual and population at large.

### Limitations

We identified obese patients based on existing dichotomous variable in NIS classifying patients as obese or non-obese. Although previous studies have used ICD-9 codes to categorize BMI, this method is not considered valid. Therefore, we could estimate adverse outcomes for subcategories of BMI. There could have been some differential reporting in NIS database. For example, variables that were not directly associated with maternal care could not have been recorded in the NIS. This could have either underestimated or overestimated the reported odds ratios. In addition, obesity being a high-risk condition, obese patients may have been closely monitored, resulting in higher estimation of their chronic conditions. Though higher reporting of chronic conditions could have occurred for patients with main outcomes in this study (labor induction, cesarean section, preeclampsia), majority of the main outcomes were conditions and procedures that are routinely evaluated during delivery hospitalizations, decreasing the chances of surveillance bias. In addition, we did not know whether obese patients were already on diet/exercise regimens because this information is lacking in the NIS. This could have affected our findings because we could not estimate the effects of these interventions on our outcomes. NIS being a deidentified database, maternal and fetal records could not be linked and hence information such as neonatal death, birth weight, and Apgar scores could not be tallied with maternal records. Since NIS is an administrative data, we did not have information on maternal weight gain during pregnancy and indications for cesarean sections. In addition, we have only included NIS data collected during 2010–2014. NIS used ICD-9 codes until 2014 and subsequently ICD-10 codes from 2015. Therefore, in order to avoid misclassification bias, we restricted our study period.

## Conclusion

Using a nationally representative database, we found that obese pregnant women had significantly greater risk for adverse obstetrical outcomes, which substantially increased the hospital and economic burden. They had greater risk for cesarean section, labor induction, pregnancy related hypertension, preeclampsia, gestational diabetes, premature rupture of membranes, chorioamnionitis, and venous thromboembolism. They also had greater risk for adverse fetal outcomes such as excessive fetal growth and fetal distress. With increasing obesity rates in the US, these adverse outcomes and associated risk factors will continue to increase in severity and numbers. Therefore, it is important to institute precautionary measures such as pre-pregnancy counseling regarding the adverse effects of obesity during pregnancy, healthy diet and exercise, lifestyle modifications, and bariatric surgeries in extreme cases. Risk stratification of pregnant patients based on obesity could also help obstetricians to make better clinical decisions and improve patient outcomes.

## Supplementary Information

Supplementary Information.
