# Supplementary material for: Obstetric outcomes during delivery hospitalizations among obese pregnant women in the United States
Source: Sci Rep. 2022 Apr 27;12:6862. doi: 10.1038/s41598-022-10786-9 (PMC9046286; doi:10.1038/s41598-022-10786-9)
Supplement: Supplementary file 1 — Supplementary Information. [file 41598_2022_10786_MOESM1_ESM.docx]

Supplementary Table 1. ICD-9-CM codes for baseline characteristics

| **Baseline characteristics** | **ICD-9-CM codes** |
| --- | --- |
| Multiple Births | V27.2-V27.7, 651.xx |
| Previous cesarean delivery | 654.2x |
| Pre-existing Diabetes Mellitus | 648.0x, 250.xx |
| Chronic Renal Disease | 581.xx-583.xx, 585., 587, 646.2x |
| Pre-existing Hypertension | 642.0x-642.2x, 642.7x, 401.x, 402.xx-405.xx |
| Depression | 296.2x, 296.3x, 300.4, 311, 298.0, 309.0, 309.1 |
| Alcohol and Substance Abuse | 291.xx, 292.xx, 303.xx-305.xx, 648.3x, 655.5x, 965.0x, V65.42 |
| Psychiatric Disorders |  |
| Psychotic Disorders | 295.xx-298.x, 293.81-293.82 |
| Mood Disorders | 293.83 |
| Anxiety Disorders | 293.84, 300.xx |
| Personality Disorders | 301.xx |
| Eating Disorders | 307.1, 307.51 |
| Adjustment Disorders | 309.24, 309.28, 309.3, 309.4, 309.9 |

Supplementary Table 2. ICD-9-CM codes for adverse maternal and fetal outcomes

| **Adverse maternal and fetal outcomes** | **ICD-9-CM codes** |
| --- | --- |
| Cesarean delivery | 74.0-74.2, 74.4, 74.99, 669.7x |
| Induction of Labor | 73.1, 73.4 |
| Pregnancy-related hypertension |  |
| Gestational hypertension | 642.3x, 642.9x |
| Preeclampsia | 642.4x, 642.5x, 642.6x, 642.7x |
| Antepartum Hemorrhage | 641.1x, 641.2x, 641.3x, 641.8x, 641.9x |
| Postpartum Hemorrhage |  |
| Due to atony | 666.1x |
| Not due to atony | 666.0x, 666.2x, 666.3x |
| Severe Postpartum Hemorrhage | [666.0x-666.3x] plus either blood transfusion [99.0x] or hysterectomy [68.3x-68.9] |
| Gestational diabetes | 648.8x |
| Preterm labor | 644.2x |
| Premature Rupture of Membranes | 658.1x |
| Chorioamnionitis | 658.4x |
| Poor Fetal growth | 656.5x |
| Excessive fetal growth | 656.6x |
| Fetal distress | 656.3x, 659.7x |
| Fetal abnormalities |  |
| Central nervous system malformations | 655.0x |
| Chromosomal abnormalities | 655.1x |
| Hereditary disease in family possible affecting fetus | 655.2 |
| Decreased fetal movements | 655.7x |
| Other/unspecified abnormalities | 655.8x 655.9x |
| Stillbirth | V27.1, V27.3, V27.4, V27.6, V27.7, 656.4x |
